# Improving Microbial Genome Annotations in an Integrated Database Context

**DOI:** 10.1371/journal.pone.0054859

**Published:** 2013-02-12

**Authors:** I-Min A. Chen, Victor M. Markowitz, Ken Chu, Iain Anderson, Konstantinos Mavromatis, Nikos C. Kyrpides, Natalia N. Ivanova

**Affiliations:** 1 Biological Data Management and Technology Center, Lawrence Berkeley National Laboratory, Berkeley, California, United States of America; 2 Microbial Genomics and Metagenomics Program, Department of Energy Joint Genome Institute, Walnut Creek, California, United States of America; Hospital for Sick Children, Canada

## Abstract

Effective comparative analysis of microbial genomes requires a consistent and complete view of biological data. Consistency regards the biological coherence of annotations, while completeness regards the extent and coverage of functional characterization for genomes. We have developed tools that allow scientists to assess and improve the consistency and completeness of microbial genome annotations in the context of the Integrated Microbial Genomes (IMG) family of systems. All publicly available microbial genomes are characterized in IMG using different functional annotation and pathway resources, thus providing a comprehensive framework for identifying and resolving annotation discrepancies. A rule based system for predicting phenotypes in IMG provides a powerful mechanism for validating functional annotations, whereby the phenotypic traits of an organism are inferred based on the presence of certain metabolic reactions and pathways and compared to experimentally observed phenotypes. The IMG family of systems are available at http://img.jgi.doe.gov/.

## Introduction

The ultimate goal of genome and metagenome analysis is the biological interpretation of the genome sequences in terms of biochemical capabilities of organisms and their niche-specific adaptations including generation of testable hypotheses about their physiological characteristics. This process entails associating genes with functional roles which describe their enzymatic activities, involvement in various macromolecular interactions and regulatory processes. These functional roles are interpreted in relation to the functional context in which these genes operate and which can be represented by pathways, ontologies or other types of functional classifications. Several genome analysis resources, such as SEED [1], MicrobesOnline [2], PATRIC [3], KEGG [4], and MetaCyc [5] support biological interpretation of microbial genomes and/or metagenomes by integrating diverse data ranging from nucleotide and protein sequences to various catalogs of protein families and functional roles, to databases of chemical compounds and reactions. Most of these resources maintain computational pipelines that assign functional roles to genes and infer the presence of reactions and pathways. Some resources, such as MicroScope [6], also support manual data curation by domain experts. Due to the diversity of data models, annotation procedures and curation techniques employed by these resources, the results of analyzing the same genome or metagenome may vary greatly between resources. Finding an explanation for these discrepancies often represents a challenge for scientists due to the use of resource-specific object identifiers (e.g., resource-specific accession numbers for protein sequences) and semantic heterogeneity. Different mapping approaches implemented in PATRIC’s (ID mapping based on genome coordinates) and M5NR database (protein sequence-based mapping) [7] facilitate the comparison of annotations provided by different resources, but do not address the problem of semantic differences.

Effective interpretation of microbial genome and metagenome data requires a consistent and complete view of biological data. **Consistency** regards the “biological coherence” of functional annotations. It is generally accepted that proteins with the “same” activity encoded by different genomes should be assigned to the “same” functional roles and associated with the “same” pathway. However, due to the dynamic and multifaceted nature of protein activity, which may be catalytic or structural, includes thermodynamic and kinetic aspects, as well as various regulatory interactions, the definition of the “same” protein activity is not straightforward. Therefore a reductionist approach is usually taken, whereby the “same” activity of the protein is defined as participation in transformations that include the same compounds (substrates, products, cofactors) or the same binding partners (small compounds or macromolecules), while leaving aside most regulatory, thermodynamic and kinetic aspects. Similar challenges exist with the definition of the “same” pathway, which depends on the larger context of an organism-specific metabolic network; however, in practice this prerequisite is usually ignored. As a result, the distinction between complete and incomplete pathways or reversible pathways operating in different directions is often lost.

**Completeness** regards the extent of functional characterization of an organism based on its genome sequence, i.e., the coverage of the “catalog” of all functional roles and pathways of an organism with the genes identified in its genome. Note that “completeness of annotation” often refers to the number of genes in a genome to which functional roles have been assigned. While the two definitions are related, there is an important difference concerning the **precision** of annotations. Although the goal of functional annotation is to assign the gene to a specific enzymatic reaction or macromolecular structure, in reality the genes are often assigned to imprecise functional roles, such as an entire class of enzymatic activities (e.g., “aminotransferase” or “short chain dehydrogenase”). Such genes will be included in assessing annotation completeness defined as the number of genes assigned to functional roles. However, in our definition of completeness, such genes will be disregarded, as these imprecise functional roles are unlikely to be part of the “function catalog” of any living organism. Annotation completeness in terms of associating genes with functions from an organism’s “function catalog” is representative of our real understanding of microbial biochemistry and genetics, but is difficult to estimate since the content of such a catalog is generally unknown. However, part of the functional repertoire of an organism can be inferred from general biological considerations, such as the necessity of housekeeping processes including DNA replication, transcription and translation, whereas another part can be deduced using metadata for genomes and metagenomes.

**Metadata** describes the important details of microbe’s lifestyle such as isolation site, habitat type, and physiological characteristics (e.g., oxygen requirements, temperature range and salinity). In addition, organism metadata often include experimentally observed phenotypes. **Phenotypes** are required for taxonomic characterization of an organism and can range from relatively simple features such as the ability to produce certain enzymes (e.g., expression of catalase or beta-glucosidase activity) to more complex characteristics, such as growth requirements (auxotrophy for certain compounds) or the type of the energy metabolism (aerobic respiration, anaerobic respiration, etc.). One of the most powerful tools for assessing the completeness and consistency of functional annotation is genome sequence-based **phenotype prediction** whereby the phenotypic traits of an organism are inferred based on the presence of certain metabolic reactions and pathways and compared to experimentally observed phenotypes. Discrepancies between predicted and observed phenotypes may require experimental verification of the predicted metabolic capacity of certain genes and proteins, as well as their expression levels and regulatory mechanisms.

Functional annotation procedures and pipelines for microbial genomes have always strived to achieve the highest possible level of annotation completeness and consistency. The exponential growth in the number of sequenced genomes and metagenomes have made this task daunting. Designing a largely automated computational functional annotation pipeline requires striking a fine balance between very precise and very specific annotations generated for a few model organisms, and the need to infer the functions of genes in less studied organisms without committing the fallacy of “over annotation.” The latter refers to the computational inference of a very precise functional role in an organism that does not have it, and is often considered even more dangerous than “under annotation”, the assignment of an imprecise functional role to a gene when a more precise role could be provided.

Best annotation practices established over the years include assignment of predicted proteins to sequence similarity-based protein families, such as COGs (Clusters of Orthologous Genes) [8] and Pfams (protein families and domains) [9]. Such assignments are usually performed by comparing protein sequences to a consensus sequence of the family represented by a Hidden Markov Model (HMM) or a Position-Specific Scoring Matrix (PSSM). Often these assignments alone are sufficient for generating very precise functional annotations for genes with largely vertical inheritance (from parent to children) and nearly non-existent duplication and neo-functionalization events, such as ribosomal proteins or subunits or RNA polymerase. In other cases assignments to COGs and Pfams generate what can be considered imprecise functional assignments, whereby a protein is assigned to a large enzymatic class or a broad functional family, such as transmembrane subunits of ABC transporters.

In most cases gene assignments to COGs and Pfams are characterized by a high degree of consistency, since the assignment method ensures detection of even remote sequence similarity provided that functionally important amino acid residues are conserved. However, the precision of COG- and Pfam-based functional annotations is generally insufficient and results in low degree of annotation completeness. In order to overcome this limitation different genome analysis resources employ a variety of manual curation procedures and computational pipelines including TIGRfam protein families [10], KEGG Orthology terms [4] and FIGfams [1]. In general, these annotation refinement tools rely on a combination of orthology detection methods (such as reciprocal best hits), sequence similarity thresholds, and analysis of synteny to assign more specific functional roles to genes. Other methods for annotation refinement are based on improved orthology detection via reconstruction of phylogenetic trees for protein families and their comparison to the species tree [11], [12], or attempt to detect subfamilies with different specificity based on the analysis of so-called Specificity Determining Positions (SDPs) [13]. However these tools may perform very well on one or a few protein families, while failing on others; it is not uncommon that different methods produce conflicting functional assignments for the same protein. Therefore careful identification of subfamilies, which may involve a combination of diverse methods, such as those described above, and manual curation of functional annotations remains a gold standard in ensuring completeness and consistency of the data.

IMG [14], [15] belongs to the collection of microbial genome and metagenome data management and analysis systems. IMG supports microbial genome interpretation by integrating diverse data from multiple sources including native annotations and native pathway collections from SEED, KEGG and MetaCyc. IMG also provides an extensive suite of tools that allow assessing the consistency and completeness of functional annotations via analysis of pathways and inferred phenotypes, as well as identification of problematic genes and protein families that may require manual curation. IMG has proved to be effective in supporting microbial genome data analysis, as illustrated by the science publications (e.g., see http://img.jgi.doe.gov/publication.html) that relied on it. The tools and mechanisms provided by IMG for improving the consistency and completeness of microbial genome annotations are described in the following sections.

## Methods

### Functional Annotations

In many cases a discrepancy between the functional assignments generated for the same gene by different resources indicates that only one (or even none) of these conflicting functional assignments is correct. IMG helps the users to identify such genes and gene families by integrating the data from different annotation sources. IMG’s annotation process [16] attempts to assign every protein-coding gene to three types of sequence-similarity based protein classifications: COG, Pfam and TIGRfam, as well as the models collected in the integrated database InterPro [17]. These protein classifications have been generated using different approaches, and protein families carry more or less extensive manually curated functional descriptions; as a result, the information provided by them is only partially redundant. A protein belonging to one COG and one TIGRfam may have several Pfam assignments corresponding to individual domains. For example, the *E.coli* rpo S sigma factor has one COG and four Pfam domains, while *E.coli* beta-glucosidase bglX has one COG and two Pfam domains. On the other hand, the genes belonging to the same COG may be assigned to different TIGRfams, since the latter classification has been designed to capture equivalogs, i.e., homologous proteins that have the same conserved function since their last common ancestor.

IMG also includes native annotations generated by other resources, such as KO terms and FIGfams. Instead of a consensus-based mechanism of functional inference, which is based on multiple sequence alignment of the proteins assigned with the same functional role and generation of the consensus sequence or a model, these systems employ pairwise sequence similarity between manually annotated seed sequences and candidate proteins, which could be assigned to this functional role. Since KO terms and FIGfams systems do not employ a consensus-based approach, there is a possibility that proteins assigned to the same functional role may belong to different sequence similarity-based protein families. For instance, KEGG Orthology term K01183 (chitinase) includes genes from glycosyl hydrolase family 18 and also those from NlpC/P60 family from Peptidase CA clan; members of these two protein families have no sequence similarity to each other. On the other hand, since both systems are based on a seed set of genes with manual assignments performed using additional refinement criteria, such as orthology detection and synteny, they often provide finer granularity of functional annotations than those generated by COGs and Pfams. Protein assignments to KO terms and FIGfams are imported from the corresponding resources using identifier- or protein sequence-based mapping, and in the case of KEGG Orthology native annotations are supplemented by additional assignments of IMG proteins to KO terms using the methodology described in [16].

**Figure 1:**
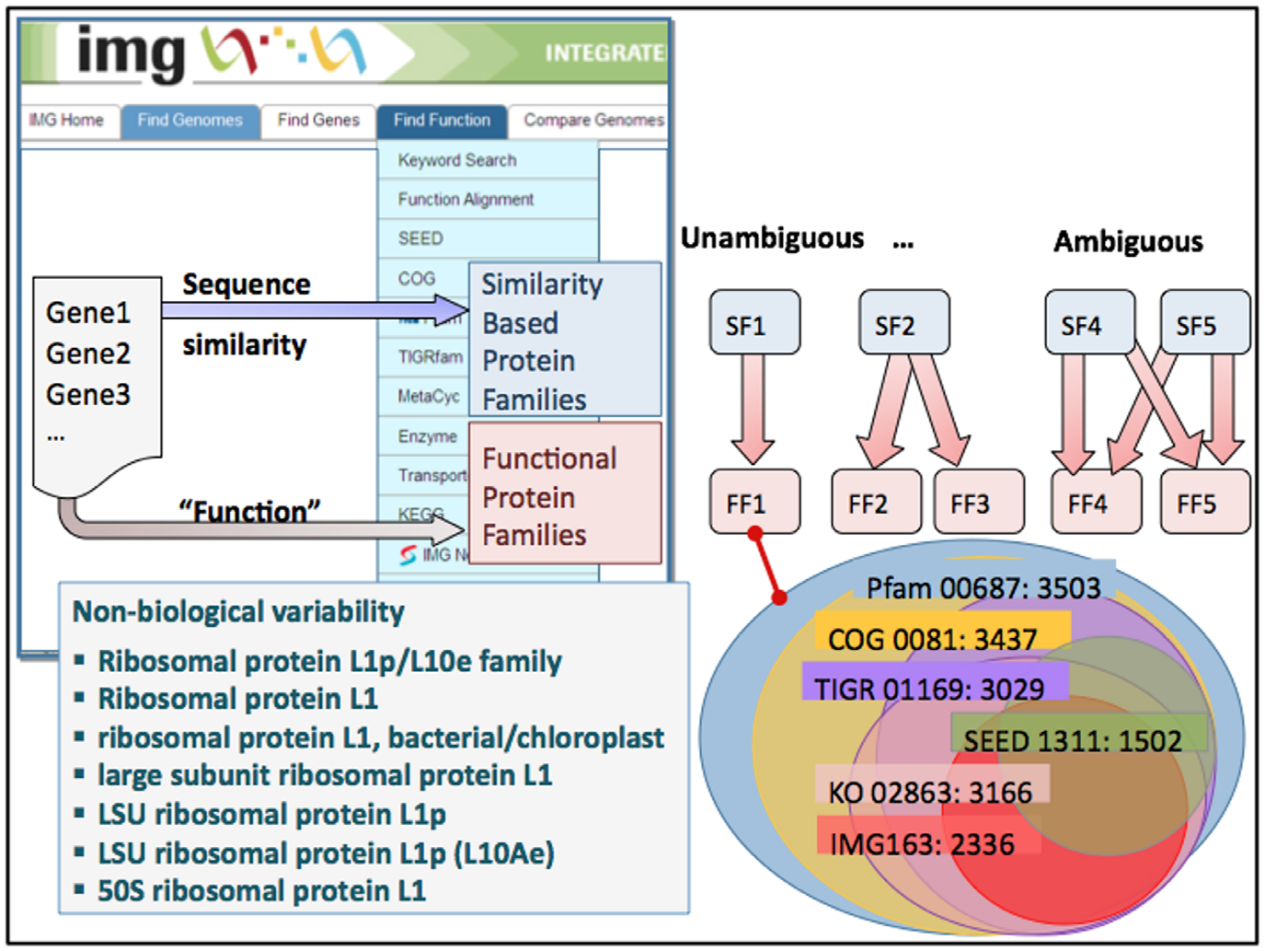
Correspondence between functional annotations in IMG using an example of ribosomal protein L1 and a combination of Pfam, COG, TIGRfam, FIGfam, IMG term and KO term annotations. The same gene may be assigned to one or more functions according to each classification system; COG, Pfam and KO make no distinction between the cytosolic and organelle forms of ribosomal protein L1, while FIGfams include more than 10 synonymous annotations for proteins with different subcellular localization, taxonomic affiliation, and subsystem membership, such as “LSU ribosomal protein L1”, “LSU ribosomal protein L1p (L10Ae)” and “Ribosomal protein L1”. The sets of proteins classified as ribosomal protein L1 by each annotation system are represented by circles of different color; the size and extent of overlap between the circles reflect the relationship between sets of proteins annotated by each protein classification system.

Combining functional annotations from different sources provides an easy way to identify annotation discrepancies, as illustrated in Figure 1. This figure shows the relationship between gene sets annotated with the same functional role – that of ribosomal protein L1 of large ribosomal subunit – by different protein classifications and annotation resources. Whereas COG and Pfam annotations generally agree and capture the majority of the genes from this functional family, annotations generated by TIGRfam, KEGG and SEED are less consistent and overlap only partially. These discrepancies can be attributed to both the selection of seed sequences and to different methodologies used to pledge new sequences to these functional roles (consensus-based approach vs. pairwise sequence similarity described above). In addition the differences in sensitivity of Pfam and TIGRfam, which employ the same model-based approach, can be ascribed to the focus of the latter on targeted subfamily models describing equivalog groups of genes resulting in rather stringent assignment cutoffs. In cases of protein families with a large number of paralogs and frequent events of gene duplication and gene loss, the discrepancies between gene sets assigned to the same functional role by different annotation resources are even more pronounced. Sometimes they are due to annotation errors, but quite often they are indicative of the presence of a subfamily with different substrate specificity (or otherwise different function), which has not been identified as such by any of the annotation resources.

**Figure 2:**
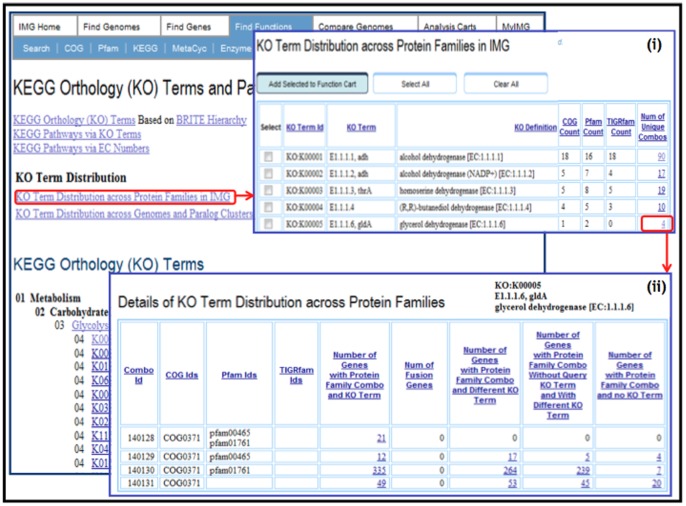
IMG tools for analysis of the correspondence between KO Terms and other protein families in IMG. (i) The summary table listing all KO terms and their correspondence to other protein families (COGs, Pfams, TIGRfams) based on the genes assigned to both; for instance, KO term K00005 (glycerol dehydrogenase) corresponds to one COG, 2 Pfams and 0 TIGRfams, with 4 unique combinations of all 3 functional assignments. (ii) Detailed lists of genes corresponding to unique combinations of functional assignments using an example of K00005; 4 unique combinations of COG and Pfam for K00005 are shown with the counts of genes assigned to this particular combination, as well as the counts of genes with this combination of COG and Pfam and different KO term assignment or no KO term assignment. In this case the majority of the genes with K00005 term are assigned to COG0371 and pfam01761; however, many genes with the same COG and Pfam are assigned to other KO terms indicating either the lack of consistency of KO term assignment or the broadness of the corresponding COG and Pfam, which may include multiple paralogous proteins with different enzymatic activities.

**Figure 3:**
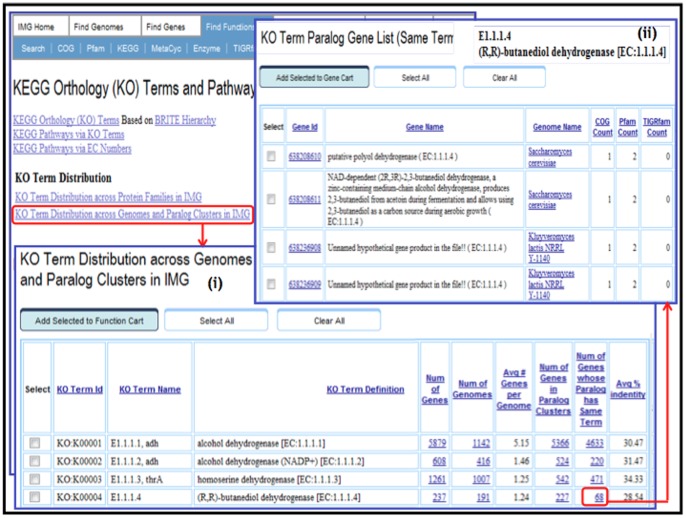
KO term distribution across genomes and paralog clusters in IMG. (i) The summary table listing all KO terms with the number of genes to which they were assigned and the number of genomes in which these genes were found, including the average number of genes with this term per genome. The higher average number of genes with the term per genome and higher number of paralogs assigned with the same term can be indicative of over-annotation. (ii) Detailed view of the paralogous genes assigned with the same KO term; as an example, 2 genes in *Saccharomyces cerevisiae* genome are assigned with the term K00004, (R,R)-butanediol dehydrogenase. One of them appears to be an experimentally verified (2R,3R)-2,3-butanediol dehydrogenase, while the other protein is a putative polyol dehydrogenase with unknown specificity.

IMG provides tools for further exploration of such subfamilies via the analysis of the chromosomal synteny and phylogenetic occurrence profiles [18], [19]. An illustration of IMG tools developed for assessment of the consistency of functional annotation is provided below, using KO terms as an example. The suite of tools includes the following:

1. The **KO Term Distribution across Protein Families**, shown in Figure 2(i), lists for each KO term the number of different COGs, Pfams, and TIGRfams it is associated with across all the genes in IMG. The number of unique (COG, Pfam, TIGRfam) combinations associated with each KO term as part of an IMG gene annotation is also provided, whereby the details for these combinations can be examined using the **Details of KO Term Distribution across Protein Families** page, as illustrated in Figure 2(ii). For a specific (query) KO term, this page lists for each unique (COG, Pfam, TIGRfam) combination:
  - the number of genes associated with the query KO term and this (COG, Pfam, TIGRfam) combination;
  - the number of genes associated with this (COG, Pfam, TIGRfam) combination and a KO term different from the query KO term, including genes associated with multiple KO terms and a query KO term as one of them;
  - the number of genes associated with this (COG, Pfam, TIGRfam) combination and a KO term different from the query KO term, and **not** associated with the query KO term;
  - the number of genes associated with this (COG, Pfam, TIGRfam) combination and not associated with a KO term.
2. The **KO Term Distribution across Genomes and Paralog Clusters**, where Paralog Clusters in IMG are determined using pairwise similarities between genes of the same genome using USEARCH, and subsequently clustered using the Markov Cluster Algorithm (MCL), lists for each (query) KO term (Figure 3(i)):
  - the number of genes associated with the query KO term;
  - the number of genomes that have genes associated with the query KO term;
  - *average number of genes* associated with the query KO term per genome; this metric helps identify KO terms that were assigned to multiple genes in the same genome, either by mistake or because these terms correspond to sequence similarity-based families rather than function-based groups;
  - the number of genes associated with the query KO term that belong to paralog clusters; the list of these genes is provided in a separate page, where one can examine the paralogs annotated with the query KO term within each genome; this metric indicates the likelihood of incorrect annotations due to the presence of paralogs.
  - the number of genes associated with the query KO term, and that have a paralog annotated with the same KO term; the list of these genes is provided in a separate page, as illustrated in Figure 3(ii), where one can examine the paralogs annotated with the query KO term within each genome; this number helps identifying incorrectly annotated paralogous genes.
  - average % identity between the paralogs annotated with the same KO term; in general, paralogs with high sequence similarity tend to have the same function, whereas, low % identity between paralogs is often a sign of significant functional divergence.

**IMG** native **functional** **terms** were introduced with the goal of addressing problems related to the consistency and completeness of functional annotations in IMG. In contrast to other types of functional annotations, IMG terms model the relationship between the protein product of a gene and its mature, fully functional form. Accordingly, three types of IMG terms have been introduced: *protein product*, corresponding to the immediate translation product of the ribosome, *modified protein*, describing the cases of covalent and non-covalent modification, such as proteolytic cleavage or cofactor binding, and *protein complex*, capturing the instances of protein-protein interaction. These terms are largely self-explanatory and help describe the transformation of a gene product into its functionally active form.

Consider an example of a multimeric enzymatic complex, such as nitrogenase, which consists of several different subunits, each of them carrying different cofactors. Oftentimes these subunits are subject to proteolytic cleavage during secretion process; in addition, maturation (e.g., cofactor binding) of one or more subunits in such complexes is not spontaneous, and requires participation of accessory proteins. Furthermore, assembly of this multimeric complex may depend on one or several chaperones, which do not participate directly in biochemical transformation catalyzed by the mature enzyme, but are required for its conversion into a catalytically active form. Examples of such enzymatic complexes are plentiful among oxidoreductases, and also can be found in other enzyme classes. Without a proper data model and accurate recording, inferring the presence of reactions catalyzed by such enzymatic complexes is quite challenging; false positive inferences are not uncommon, in most cases based on the presence of a gene with similarity to just one subunit (not necessarily a catalytic one). Although this model does not describe all the circumstances surrounding the transformation of translation products into their catalytically or physiologically active forms, IMG does provide a mechanism for capturing the most common scenarios and identification of the majority of the genes necessary for the presence of various enzymatic activities including all subunits, accessory proteins, as well as entire pathways necessary for biosynthesis of cofactors (see, for instance, pathways for nitrate reductase maturation).

Initial assignment of genes to IMG terms is done manually. Each IMG term is functionally coherent and is not required to contain sequences that share similarity. Subsequently the IMG terms are propagated using strict sequence similarity thresholds [16] and are inspected by expert curators. IMG terms are further connected to entities describing transformations of various metabolites and macromolecules called **IMG reactions** and **IMG pathways**, as described below.

### Pathway Annotations

IMG integrates pathway information from multiple sources including KEGG Pathway Maps and Modules, BioCyc/MetaCyc pathways, MPW pathways [20], SEED subsystems, and IMG native pathways. These resources describe metabolic transformations catalyzed by proteins as well as some other reaction types, such as protein-protein interactions. These resources provide a good representation of core microbial processes and, to some extent, of the variable part of microbial metabolism, thus helping to establish the context in which proteins encoded by the genome are expected to function.

Although most pathway collections include the same set of core biosynthetic pathways commonly found in living organisms (e.g., glycolysis, amino acid and nucleotide biosynthesis), different resources focus on different aspects of biochemical reactions and different facets of metabolism. Furthermore, due to the inconsistency of data models and pathway recording procedures, mapping of identical or similar biochemical transformations between different pathway resources is far from straightforward. These challenges provide the motivation for integrating diverse pathway information into IMG with the goal of supporting manual exploration of genomes in the context of pathway data. The **All Pathways Search** tool allows identifying KEGG, MetaCyc and MPW pathways using EC and/or keyword matches, and thus serves as a starting point for comparing various pathways in IMG.

The native **IMG pathways** have been developed to address weaknesses of data models found in other pathway resources, such as inconsistent representation of protein complexes as reaction catalysts and alternative implementations of reaction catalysts. An IMG pathway is defined as a set of sequential generalized transformations (IMG reactions) connecting two branching points in the metabolic network or in the network of other types of reactions. In addition to the traditional metabolic reactions recording biochemical transformations of simple molecules, IMG reactions may describe transformations of complex polymers (e.g., cellulose breakdown into shorter chains), protein-protein interactions and even topological changes (e.g., unwinding of double-stranded DNA).

In order to simplify pathway recording, alternative transformations of the same metabolites (e.g., decomposition of hydrogen peroxide by peroxidases with different cofactor specificities) are allowed to be merged into one IMG reaction, provided that the directionality of transformation is the same. Each IMG reaction is associated with one or more IMG terms, which can participate in the reaction as catalysts, or as substrates and products (e.g., proteolytic cleavage of an inactive precursor producing a mature enzyme). During the genome annotation process, genes in the genome are assigned to IMG terms using a standard operating functional procedure [16]. Briefly, a gene is assigned to an IMG term if it has at least five homologs in the IMG database with >50% identity and at least two of these five homologs have an IMG term assigned, and the alignment length of the pairwise comparison should be >70% of the length of both the query and target proteins. These automated assignment criteria were developed to minimize the possibility of false positive assignments (over-annotation).

An IMG reaction is considered to be present in an organism if the latter has the genes assigned to all IMG terms that are necessary for the reaction to proceed. In the case of IMG reactions including alternative transformations, only one of the alternatives needs to be present; on the other hand, if an IMG reaction is catalyzed by a protein complex, the presence of all subunits is required. The same rules apply not only to the traditional metabolic reactions, but also to protein-protein interactions and topological transformations. For instance, the reaction of “Replicative DNA helicase loading” has two alternative implementations: helicase loading through ring-breaking or ring-making mechanism. Only one of these alternatives needs to be present in order to consider this IMG reaction to be present in an organism.

Similar to IMG reactions, the presence of IMG pathways in an organism can be inferred based on the presence of IMG reactions: if this requirement is fulfilled, the presence of IMG pathway in an organism is *asserted*. In addition to the automated pathway assertion using the set of rules described above, domain experts can manually *assert* an IMG pathway even in the absence of a full complement of genes necessary for all IMG reactions or mark the pathway “absent” even in the presence of some genes. These manual options are especially useful for the analysis of incomplete genome sequences and reduced genomes of intracellular symbionts and pathogens.

Since IMG pathways serve as building blocks for IMG phenotypes (see below), two additional status values exist for IMG pathway assertion. IMG pathways without a full complement of the genes that were not *asserted* manually can have the status of “*unknown*” or “*not asserted*”. The status “*unknown*” is assigned to an IMG pathway, in which IMG terms without gene assignments have “candidate genes”. The latter are defined as those with bi-directional best BLASTp hits (BBH) to the genes in other genomes that are assigned to the corresponding IMG term. Such a candidate gene can be associated with an IMG term if at least two of its top 5 BBH genes are assigned this term, sequence overlapping is over 70%, and at least one of these BBH genes has percent identity greater than 25%. In most cases “candidate genes” are not assigned to the corresponding IMG term because of the alignment between the “candidate gene” and its homologs assigned with the term does not satisfy rather stringent criteria for automated IMG term assignment. Such genes can be manually inspected by the domain experts and manually assigned to the IMG term; however, even in the absence of such assignment, it is important to distinguish between the genomes with and without candidate genes. The presence of “candidate genes” is considered sufficient evidence that the pathway in which the corresponding IMG term participates may be present in an organism resulting in an assertion status of “unknown”. In contrast, the status “*not asserted*” is assigned to an IMG pathway when no “candidate genes” can be found. This 3-value logic implemented in IMG pathway inference distinguishes IMG pathways from other pathway resources, which consider only 2 status values, “present” and “absent.” It helps to minimize false positive and false negative inferences by distinguishing “*asserted*” pathways, which have a full complement of genes and therefore highly likely to be present in an organism, from those that may be present (assertion status of “*unknown*”) and those that are unlikely to be present (assertion status of “*not asserted*”).

For example, 6-phosphogluconate synthesis via gluconate pathway (P_339_) is specified as a sequence of 3 reaction steps. The first step is glucose dehydrogenase or glucose oxidase, which can be defined as any one of the following four alternative IMG reactions:

- R_977_: D-glucose+NADP+< = >D-glucono-1,5-lactone+NADPH+H+
- R_978_: D-glucose+NAD+< = >D-glucono-1,5-lactone+NADH+H+
- R_979_: D-glucose+O2< = > D-glucono-1,5-lactone+H2O2
- R_980_: D-Glucose+Ubiquinone< = >D-Glucono-1,5-lactone+Ubiquinol

Note that although these reactions utilize different coenzymes (NADP+, NAD+, etc.), they all convert D-glucose into D-glucono-1,5-lactone with the same anomeric specificity towards beta-D-glucose; therefore these reactions will be included into the same pathway as alternative implementations of conversion of beta-D-glucose to D-glucono-1,5-lactone.

The second step is gluconolactonase, which can be defined by the following IMG reaction:

- R_981_: D-Glucono-1,5-lactone+H2O< = >D-Gluconic acid

The third step is gluconate kinase, which can be defined by the following IMG reaction:

- R_982_: ATP+D-Gluconic acid< = >ADP +6-Phospho-D-gluconate

Using a rule-based representation, we have:

(R_977_ or R_978_ or R_979_ or R_980_) and R_981_ and R_982_ −>P_339._

**Figure 4:**
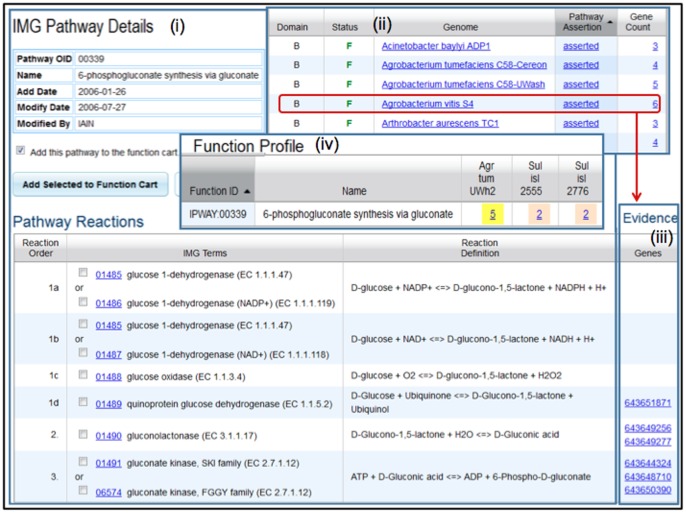
IMG Pathway Definitions and Assertions. (i) **IMG Pathway Details** displays information on a specific IMG pathway including the author and editing history, as well as the component reactions and functional roles. Assertion details for an IMG Pathway can be examined via (ii) the list of genomes for which the pathway is asserted, and (iii) the number of genes in the genome assigned to the functional roles in the pathway. Genomes can be compared in terms of IMG pathways using **Function Profile** analysis tool, with the result (iv) displaying counts of genes assigned to functional roles in the pathway.

Figure 4(i) shows the IMG pathway definition of 6-phosphogluconate synthesis via gluconate pathway. This pathway is asserted for *Agrobacterium vitis* S4, because this finished genome has all the required reactions, as illustrated in Figures 4(ii) and 4(iii). The same pathway has the status “not asserted” for draft genomes of *Rhizobium etli Brasil 5* and *Rhizobium etli GR56*, because these fragmented genomes do not have genes for some of the required reactions.

## Results

IMG terms, reactions and pathways help address problems related to the consistency and completeness of functional and pathway annotations in IMG. Thus, IMG provides users with different ways of reviewing pathway assertions:

1. Users can select a pathway to view **IMG Pathway Details**, as illustrated in Figure 4(i). In addition to the summary of IMG reactions included in the pathway, this page displays a list of all or user-selected genomes and the status of pathway assertion in the genome of interest, as illustrated in Figure 4(ii). By clicking on Pathway Assertion status (*asserted*, *not asserted* or *unknown*), users navigate to the **IMG Pathway Assertion Details** page, which shows assignments of genes to the corresponding IMG terms and IMG reactions, as illustrated in Figure 4(iii).
2. Users can explore the assertion status for multiple IMG pathways using the **Function Profile** tool. After IMG pathways and genomes of interest are selected, this tool will display an **IMG Pathways by Genomes** matrix as illustrated in Figure 4(iv), with each cell in the matrix showing the number of genes in a genome associated with the corresponding IMG pathway.

Pathways can be best understood in context of other pathways within the organism. For example, if an organism degrades cellulose to cellobiose outside the cell, it can only utilize cellulose as carbon source if it also has a transport pathway for uptake of cellobiose and, within the cell, a metabolic pathway to gain energy from cellobiose. If all steps are present, then the organism will have the **phenotype** of cellulose utilization with cellobiose as an intermediate. Phenotypes correspond directly to biological traits that can be measured in an experiment, and thus provide a powerful mechanism for further assessing the coherence of functional and pathways annotations.

**Figure 5:**
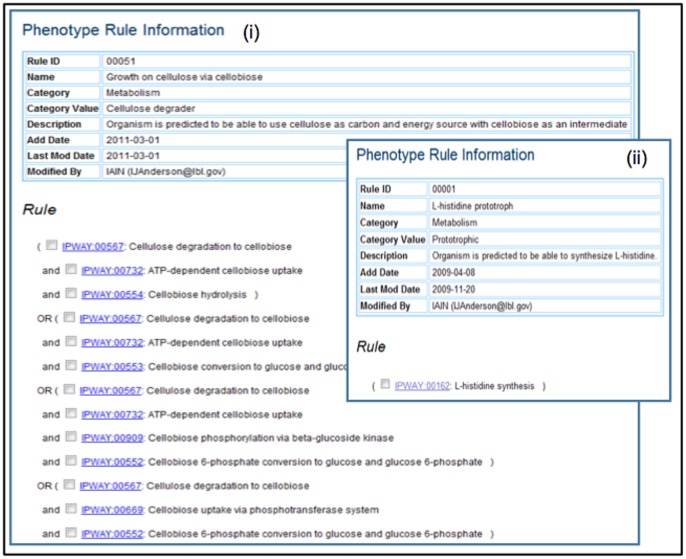
Phenotype Prediction Rules in IMG are defined as AND-OR rules based on IMG pathway assertions. Phenotype rules range from complex, such as (i) the rule for predicting “Growth on cellulose via cellobiose” which involves multiple IMG pathways, to very simple, such as (ii) the rule for predicting “L-histidine prototroph” which involves one IMG pathway.

IMG pathways provide the context needed for predicting phenotypes within the IMG system. For example, for the transport step in the cellulose utilization via cellobiose there are two known possibilities: cellobiose can be taken up by an ABC transporter or by the phosphotransferase system. Within the cell, cellobiose can either be converted to glucose and glucose 6-phosphate, glucose and glucose 1-phosphate, or two molecules of glucose. So the phenotype of cellulose utilization can be specified in IMG using the following set of logical **rules** reflecting different combination of pathways, as illustrated in Figure 5(i):

1. Cellulose degradation to cellobiose AND ATP-dependent cellobiose uptake AND Cellobiose hydrolysis; or
2. Cellulose degradation to cellobiose AND ATP-dependent cellobiose uptake AND Cellobiose conversion to glucose and glucose 1-phosphate; or
3. Cellulose degradation to cellobiose AND ATP-dependent cellobiose uptake AND Cellobiose phosphorylation via beta-glucoside kinase AND Cellobiose 6-phosphate conversion to glucose and glucose 6-phosphate; or
4. Cellulose degradation to cellobiose AND Cellobiose uptake via phosphotransferase system AND Cellobiose 6-phosphate conversion to glucose and glucose 6-phosphate.

Following this approach we have developed a system for recording IMG phenotype definitions and **inferring** organism phenotypes based on these rules and pathway assertion status. In general, IMG phenotype definitions consist of IMG pathway status values connected by logical operators AND, OR and NOT. The simplest IMG phenotype definition consists of one IMG pathway status value without operators. Consider the example of *histidine biosynthesis*, for which only one pathway is known. In this case the phenotype of *L-histidine prototrophy* (i.e., organism’s ability to synthesize histidine) can be recorded simply as the presence of the corresponding pathway (IMG pathway 162), as illustrated in Figure 5(ii). In contrast, the phenotype of *L-histidine auxotrophy* (i.e., organism’s inability to synthesize histidine) is recorded as NOT (IMG pathway 162). Given the 3-value status of IMG pathways (“*asserted*”, “*unknown*”, “*not asserted*”), evaluation of the statement NOT (IMG pathway 162) is performed as shown in Table 1 [21].

**Table 1:** NOT (3-valued logic).

IMG Pathway assertion	Evaluation result
Asserted	False
Not Asserted	True
Unknown	Unknown

Based on this evaluation an organism will be assigned the IMG phenotype of *L-histidine auxotroph* if IMG pathway 162 has the status “*not asserted*”, whereas an organism in which the status of IMG pathway 162 is “*asserted*” will be assigned the IMG phenotype *L-histidine prototroph*. Both phenotype predictions can be readily tested by attempting to grow an organism on a medium with and without L-histidine: while L-histidine prototrophs will successfully grow on both media, L-histidine auxotrophs should fail to grow in the absence of externally provided histidine. For IMG phenotypes consisting of two pathway status values connected by operators OR or AND, evaluations are performed as shown in Tables 2 and 3.

**Table 2:** OR (3-valued logic).

Evaluation result	Pathway 2: Asserted	Pathway 2: Not Asserted	Pathway2: Unknown
Pathway 1: Asserted	True	True	True
Pathway 1: Not Asserted	True	False	Unknown
Pathway 1: Unknown	True	Unknown	Unknown

**Table 3:** AND (3-valued logic).

Evaluation result	Pathway 2: Asserted	Pathway 2: Not Asserted	Pathway2: Unknown
Pathway 1: Asserted	True	False	Unknown
Pathway 1: Not Asserted	False	False	False
Pathway 1: Unknown	Unknown	False	Unknown

More complex phenotypes can be recorded using multiple pathway status values connected by multiple AND, OR and NOT operators, whereby the result of each operation is evaluated as described above. For example, in order to be a phenylalanine prototroph an organism must synthesize chorismate and then synthesize phenylalanine from chorismate. For both of these steps, there are two possible pathways, so the phenotype *L-phenylalanine prototroph* is an AND-rule that can be recorded using IMG pathway identifiers as: (IMG pathway 146 OR IMG pathway 519) AND (IMG pathway 272 OR IMG pathway 147). Another example of a complex phenotype is *L-lysine prototrophy*, which requires the presence of at least 1 out of 6 possible biosynthetic pathways, and can be recorded as (IMG pathway 169 OR IMG pathway 170 OR IMG pathway 0171 OR IMG pathway 199 OR IMG pathway 333 OR IMG pathway 465). On the other hand, the phenotype of *L-lysine auxotrophy* is recorded as (NOT IMG pathway 169) AND (NOT IMG pathway 170) AND (NOT IMG pathway 0171) AND (NOT IMG pathway 199) AND (NOT IMG pathway 333) AND (NOT IMG pathway 465).

**Figure 6:**
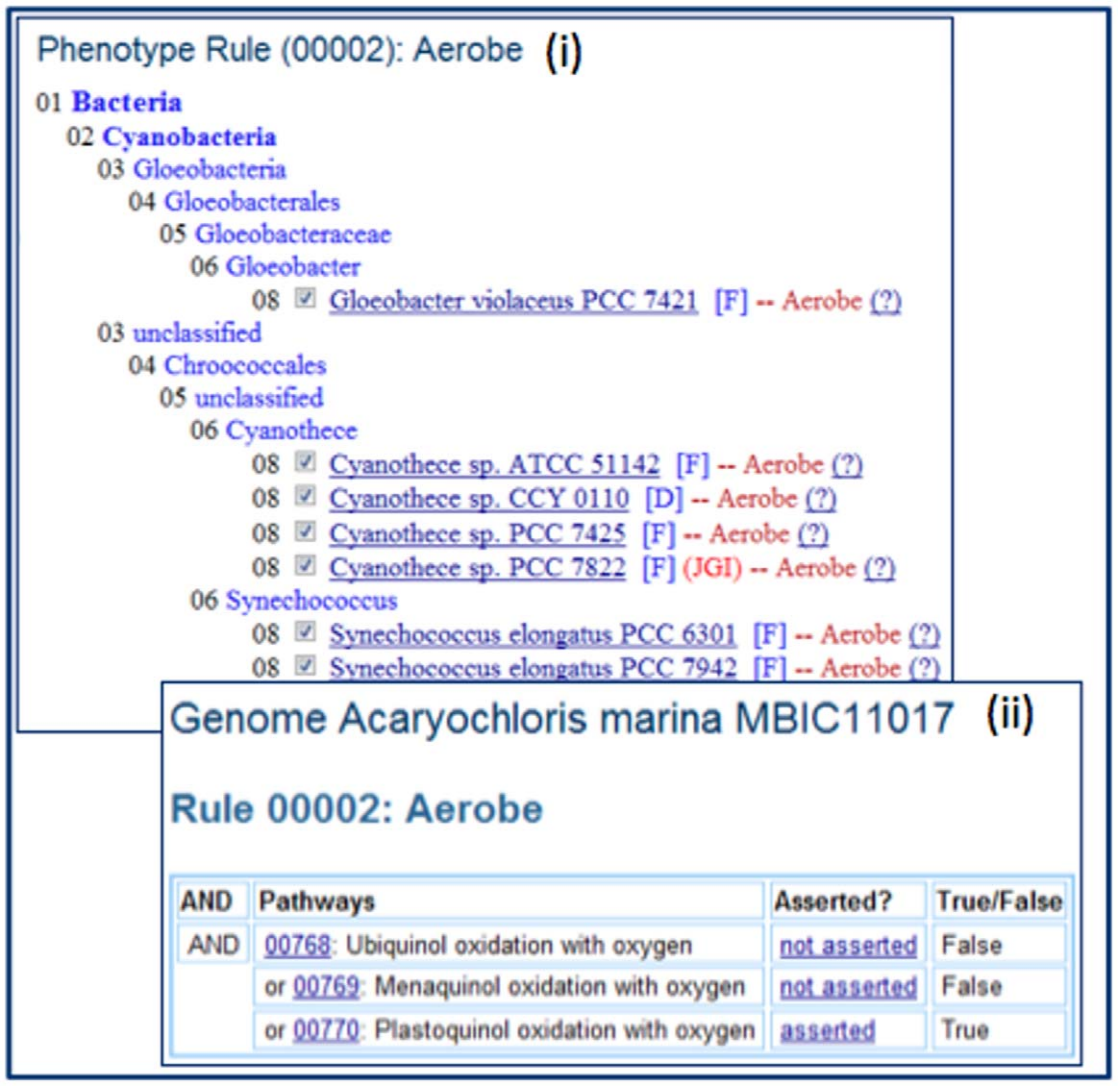
Examining phenotype prediction results in IMG. (i) All organisms that are predicted to be aerobic can be examined using a phylogenetic tree display; by clicking on “(?)”, (ii) users can examine the evidence of phenotype prediction of each organism.

IMG users can view all genomes with predicted phenotype in a table or phylogenetic tree hierarchical display. Viewing phenotype prediction using a hierarchical display has the advantage of showing the distribution of phenotypes in genetically related organisms. There are two hierarchical display options. The first display option only shows organisms with the predicted phenotype, which is more compact and easier to view, as illustrated in Figure 6(i). The second display option shows all organisms; genomes with predicted phenotypes are selected and shown with the phenotype label. This hierarchical display allows users to compare closely related organisms with and without the predicted phenotype. In both display modes, users can click on the question mark (?) to see phenotype prediction evaluation. For example, Figure 6(ii) shows the result of phenotype evaluation for *Acaryochloris marina MBIC 11017*, which has been predicted to be aerobic because “Plastuquinol oxidation with oxygen” (IMG Pathway 00770) is present in the organism.

## Discussion

The IMG tools and mechanisms presented in this paper allow scientists to review and improve the consistency and completeness of functional and pathways annotations in the integrated context of all publicly available microbial genomes. Functional and pathways annotations across all genomes can be examined in order to detect problematic (missing or inconsistent) annotations. IMG native functional terms and pathways are used to address annotation problems and provide the context needed for predicting phenotypes. Functional and pathways annotations are further validated by comparing predicted phenotypes to experimentally verified phenotypic traits of organisms.

**Figure 7:**
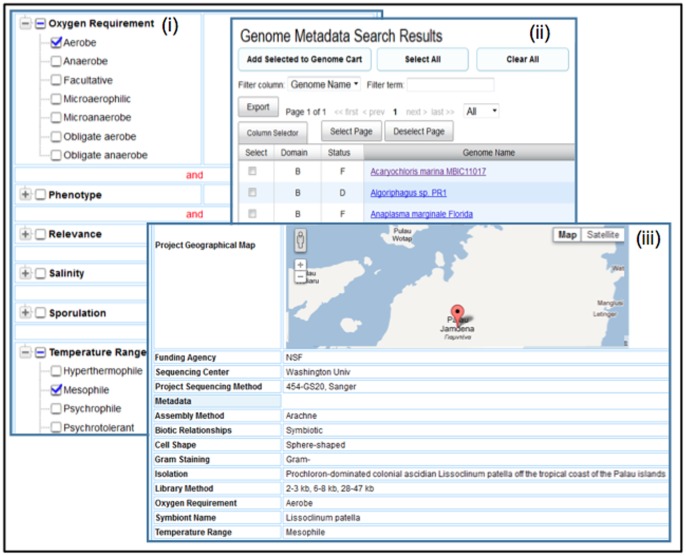
Metadata search and display in IMG. (i) Metadata search with condition “aerobe with mesophile temperature range” returns (ii) a list of genomes that satisfy this condition; following the genome name hyperlink (iii) users can further examine organism detail and associated metadata information.

Phenotypic traits of sequenced organisms are available in resources such as GOLD [22]. Genome metadata from GOLD is incorporated into IMG and can be used for reviewing predicted phenotypes. For example, one could find all aerobic, symbiotic organism with mesophile temperature range using IMG’s **Genome Search by** **Metadata** tool, as illustrated in Figure 7(i), where the search involves metadata categories Oxygen Requirement and Temperature Range. Such searches return list of genomes, as illustrated in Figure 7(ii). For each genome, the Organism Details page lists all the associated metadata, as illustrated in Figure 7(iii). In the example shown in Figure 7(iii) metadata from GOLD confirms that this organism is indeed with oxygen requirement: aerobic.

In order to facilitate the comparison of metadata from GOLD and IMG phenotype predictions, each IMG phenotype rule is associated with a *category* (which corresponds to a GOLD attribute) and a *category value* (which corresponds to a GOLD attribute value). For example, the “Aerobe” phenotype rule belongs to category “Oxygen Requirement.” Several categories of IMG Phenotypes have been defined so far, with only metabolism and oxygen requirement have been populated with phenotype rules. The most recent release of the IMG system includes 65 phenotype rules, with the number of such rules continuously extended.

It is worth noting that resources such as SEED employ stoichiometric modeling and flux balance analysis for predicting phenotype [23]. However a rule-based approach for phenotype prediction such as that followed in IMG has the advantage of allowing phenotype inferences based on partial genome sequences, such as those generated by single cell genomics [24] or metagenome binning.
